# Investigation and Manipulation of Different Analog Behaviors of Memristor as Electronic Synapse for Neuromorphic Applications

**DOI:** 10.1038/srep22970

**Published:** 2016-03-14

**Authors:** Changhong Wang, Wei He, Yi Tong, Rong Zhao

**Affiliations:** 1 Engineering Product Development Singapore University of Technology and Design 8 Somapah Road, Singapore 487372

## Abstract

Low-power and high-density electronic synapse is an important building block of brain-inspired systems. The recent advancement in memristor has provided an opportunity to advance electronic synapse design. However, a guideline on designing and manipulating the memristor’s analog behaviors is still lacking. In this work, we reveal that compliance current (*I*_comp_) of electroforming process played an important role in realizing a stable analog behavior, which is attributed to the generation of conical-type conductive filament. A proper *I*_comp_ could result in a large conductance window, good stability, and low voltage analog switching. We further reveal that different pulse conditions can lead to three analog behaviors, where the conductance changes in monotonic increase, plateau after initial jump, and impulse-like shape, respectively. These behaviors could benefit the design of electronic synapse with enriched learning capabilities. This work will provide a useful guideline for designing and manipulating memristor as electronic synapses for brain-inspired systems.

Developing brain-inspired computer has been an ultimate goal for scientists to pursue for decades^1^,^2^. It is widely accepted that synapses are responsible for the massive parallelism and structural plasticity in neurobiology, and crucial to biological computations that underlie perception and learning^3^,^4^. Therefore, electronic synaptic devices that could display the functionalities of neural synapse such as plasticity and learning are the most important building blocks of brain-inspired computer and neuromorphic systems. Electronic synapse traditionally is designed based on complementary metal-oxide-semiconductor (CMOS) technology, which is impossible to be scaled up due to high power consumption and low density, let alone reach the human brain’s level at 100 trillion of synapses^4^,^5^. The recent advancement in memristor has provided a promising opportunity to advance the electronic synapse design, which is attributed to the unique properties of memristor including analog behavior, plasticity, non-volatile, nanoscale size, and low power^6^,^7^,^8^,^9^,^10^. This has sparked a new wave of enthusiasm in developing brain-inspired computer and neuromorphic systems. To achieve this goal, one of the keys is to develop a memristor with robust and low power analog behaviors and learning capabilities, which can be scaled up to an ultra large system. Even though many memristive devices have been demonstrated by using different materials (e.g. metal oxides like SiO_2_, WO_x_, InGaZnO, chalcogenides like Cu_2_S, Ag_2_S, and phase change materials like Ge_2_Sb_2_Te_5_)^11^,^12^,^13^,^14^,^15^,^16^,^17^,^18^, they are mostly developed in an exploration way. A guideline on designing and manipulating the memristor’s analog behaviors, which is much crucial and necessary for the synapse and neuromorphic engineering, is still lacking yet. Furthermore, because the biological learning rules are diverse in nature, memristor displaying different analog behaviors is greatly needed to enhance the learning capabilities of electronic synapse.

In this work, we investigate the effect of compliance current (*I*_comp_) of electroforming process on forming the analog resistive switching of memristor using iron oxide (FeO_x_) based memristor. FeO_x_ was selected as the active layer due to the good repeatability and consistency of the devices^4^. It is found that *I*_comp_ dramatically affects the analog resistive switching of memristor in terms of conductance window, operational voltage, and stability. A conductive filament model is proposed to explain the observed analog behaviors, and the conduction mechanism of FeO_x_ based memristor is also discussed. Moreover, the analog behaviors are found to be further affected by pulse conditions. Three kinds of analog behaviors with different conductance changing trends (i.e. monotonic increase, plateau after initial jump, and impulse-like change) are observed, which can be utilized to enrich the learning capabilities of electronic synapse. These findings could provide a useful guideline for designing memristor devices as electronic synapses with comprehensive learning capabilities for brain-inspired computing systems.

## Results and Discussion

**Figure 1:**
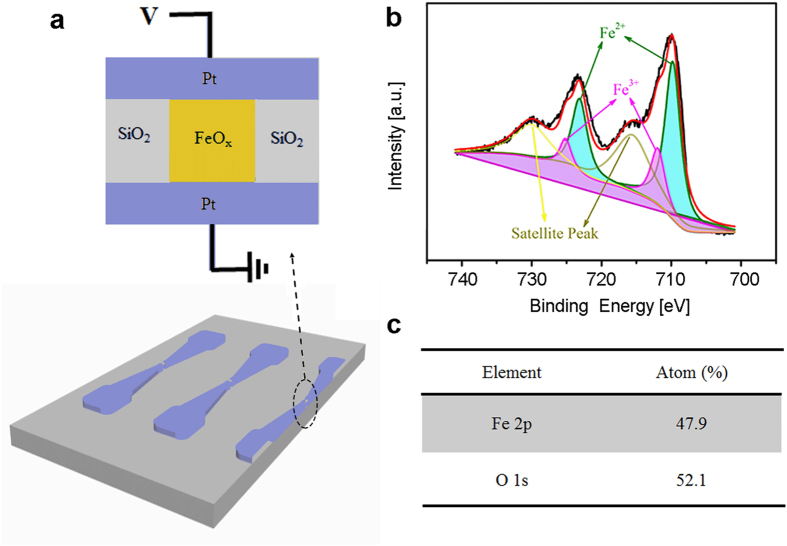
Device Characterizations. (**a**) Schematic illustration of the FeO_x_ based memristor devices on silicon wafer (bottom) with the cross-sectional view of the structure and the testing configuration (upper). (**b**) X-ray photoelectron spectroscopy (XPS) spectrum for FeO_x_ thin film, and (**c**) The atomic ratio of FeO_x_ film detected by XPS.

The FeO_x_ based memristor was fabricated on silicon wafers with 1 μm thick SiO_2_ layer on top. A schematic drawing of the cross-sectional view and top view of the device structure is shown in Fig. 1(a). A layer of 30 nm FeO_x_ film was deposited by radio frequency (RF) magnetic sputtering, which was sandwiched by two inert platinum (Pt) electrodes to form a Pt/FeO_x_/Pt structure. The details can be found in the section of experimental method. X-ray photoelectron spectroscopy (XPS) measurement was performed to analyze the chemical composition of the as-deposited FeO_x_ film (Fig. 1(b)). The peaks at 709.77 eV and 723.02 eV correspond to Fe^2+^ 2p_3/2_ and Fe^2+^ 2p_1/2_, respectively. The peaks at 712.40 eV and 725.05 eV correspond to Fe^3+^ 2p_3/2_ and Fe^3+^ 2p_1/2_, respectively. The peaks at 715.33 eV and 729.89 eV represent two satellite peaks^4^,^19^,^20^. Since the relative peak intensity of Fe^2+^ is larger than that of Fe^3+^, the deposited FeO_x_ layer is mainly composited of Fe^2+^, which is consistent with the atomic ratio of Fe and O in the FeO_x_ film (Fe:O = 1:1.09) as shown in Fig. 1(c). The X-ray diffraction patterns shown in Fig. S1 further confirm that the as-deposited FeO_x_ film is at amorphous state^21^.

**Figure 2:**
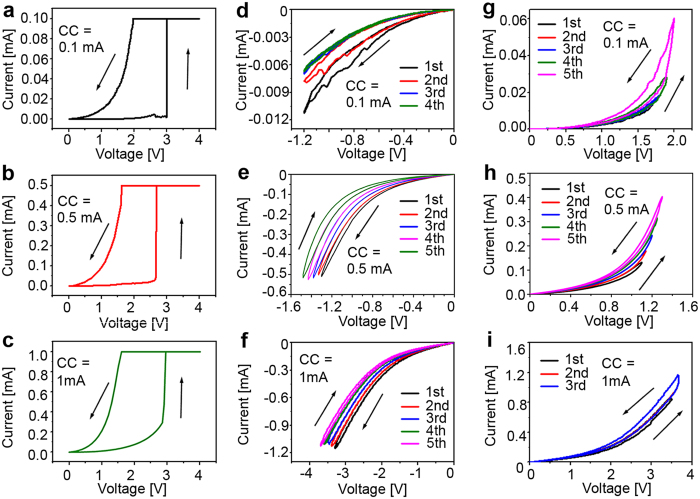
Electroforming process with different compliance current (*I*_comp_) and its consequent analog behaviors. For *I*_comp_ of 0.1 mA, (**a**) electroforming profile, (**d**) and (**g**) the analog *I*-*V* curves under negative and positive sweep voltage, respectively. For *I*_comp_ of 0.5 mA, (**b**) electroforming profile, (**e**) and (**h**) the analog *I*-*V* curves under negative and positive sweep voltage, respectively. For *I*_comp_ of 1 mA, (**c**) electroforming profile, (**f**) and (**i**) the analog *I*-*V* curves under negative and positive sweep voltage, respectively.

To study the electroforming process, *I*_comp_ with different values was applied to form the pristine FeO_x_ based memristors. All the memristor devices were at the low conductance state (~10^−7^ mS) initially. Figure 2(a–c) show the electroforming profile with *I*_comp_ of 0.1 mA, 0.5 mA, and 1 mA, respectively. After electroforming process, it can be seen that the conductance of the memristor was increased to ~10 mS, ~70 mS, and ~ 225 mS, for *I*_comp_ of 0.1, 0.5, and 1.0 mA, respectively. The reset process was conducted subsequently using direct current (DC) sweeping for devices under various *I*_comp_. As shown in Fig. 2(d–f), the conductance of all devices gradually decreases while increasing the stopping voltage of negative sweep. After that, set process was subsequently conducted as shown in Fig. 2(g–i). It is observed that the conductance of the devices increases with the increase of the stopping voltage of positive sweep.

After electroforming with three different *I*_comp_, the analog behaviors of memristor were investigated and compared. For small *I*_comp_ with a value of 0.1 mA, after electroforming, the subsequent reset/set process was able to occur at small stopping voltage (i.e. −1.20 V and 1.70 V) with small conduction current (i.e. −0.010 mA and 0.014 mA) as shown in Fig. 2(d,g). In addition, it is noticed that there are some fluctuations and overlaps between each pinched-hysteresis *I*-*V* loop, this suggests that the conductive filament (CF) formed by the electroforming process with a small *I*_comp_ of 0.1 mA is unstable and the self-degradation of CF occurs which contributes to the overlaps of pinched-hysteresis *I-V* loop. After electroforming by the increased *I*_comp_ of 0.5 mA (Fig. 2(b)), the subsequent reset/set current increased to −0.5 mA and 0.4 mA (Fig. 2(e,h)), respectively. Comparing to the analog behavior of small *I*_comp_ of 0.1 mA, there is no fluctuation observed between each *I*-*V* loop, indicating a strong CF formed by an increased *I*_comp_ of 0.5 mA. The slightly smaller set voltage in Fig. 2(h) compared to that in Fig. 2(g) may be due to different dynamics of the CF induced by different amount of oxygen vacancies^22^,^28^,^38^. Next, when we further increased *I*_comp_ to a large value of 1.0 mA, the conductance of memristor kept increasing to 225 mS as shown in Fig. 2(c). The corresponding reset/set process only occurred at very large stopping voltage (i.e. −3.5 V and 3.5 V) with large conduction current (i.e. −1.1 mA and 0.9 mA) as shown in Fig. 2(f,i). Based on the above observations, a clear trend is observed that larger *I*_comp_ causes stronger and more stable CF during the operation of FeO_x_ based memristor. Moreover, we calculated the conductance change ∆*G* which is defined as *G*_int _− *G*_fin_, where *G*_int_ is the initial conductance, and *G*_fin_ is the final conductance after the voltage sweeping. It can be noted that the largest ∆*G* is achieved for moderate *I*_comp_ of 0.5 mA. Therefore, a proper *I*_comp_ in electroforming process is critically needed for achieving good analog performance of memristors.

**Figure 3:**
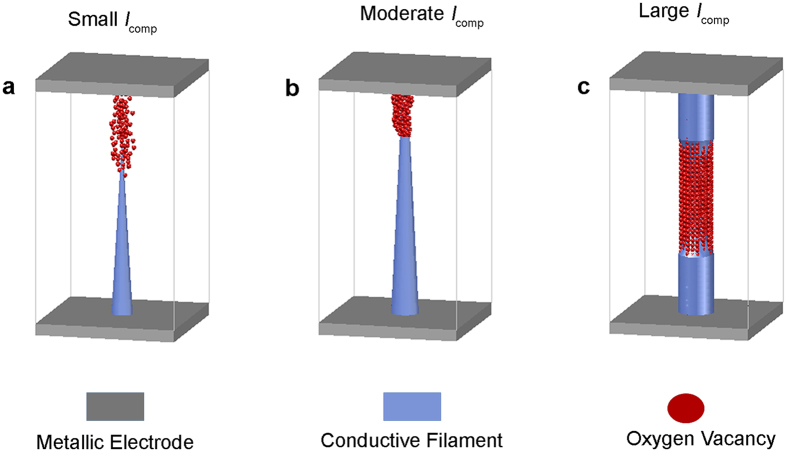
Conductive Filaments (CFs) generated by different *I*_comp_. (**a**) The pyramidal CF formed by the small *I*_comp_. (**b**) The conical CF formed by the moderate *I*_comp_. (**c**) The cylindrical CF formed by the large *I*_comp_.

Nowadays, it is widely accepted that the initial electroforming process is related to the generation of defects (e.g. oxygen vacancies) within the transition oxides, which are responsible for constructing CF within the oxides during the electroforming process^23^,^24^,^25^,^26^,^27^. Our findings disclose that the *I*_comp_ of the electroforming process would significantly affect the formation of CF and its dynamics, which consequently determine the following analog behavior. When *I*_comp_ is small, the formed CF is most likely in a pyramidal shape, as illustrated in Fig. 3(a). It is narrow due to less oxygen vacancies for constructing CF under small compliance current, which is the possible reason for the overlap and fluctuation in the *I*-*V* loops in Fig. 2(d,g). Normally, resistive switching (RS) occurs at the tip of CF because oxygen vacancies are easily driven by electric field and joule heating at this location^28^. When *I*_comp_ increases, more oxygen vacancies are generated such that a conical CF could be formed as shown in Fig. 3(b). Comparing to the case of small *I*_comp_, the size of CF should increase in both length and width, resulting in no overlap in the *I*-*V* loops in Fig. 2(e,h). The increase of size of CF has been investigated by theoretical simulation and confirmed by *in-situ* transmission electron microscopy (TEM) observation^27^,^29^,^30^. The rupture and combination of conical filament, which is the most common type of CF in memristors, normally occurs at the top of the conical filament, leading to a favorable memory window^8^,^27^,^29^,^31^,^32^,^33^,^34^. When a very large *I*_comp_ is applied to memristor, the filament will further broaden and lengthen, resulting in a high conductance which is consistent with the electrical results extracted from Fig. 2(c). The filament may become a cylinder shape as shown in Fig. 3(c). This kind of cylindrical filament tends to display digital switching but not analog switching^26^,^35^. RS in this case often occurs at the middle of the cylindrical filament, which is caused by the higher temperature at that location due to the metal electrodes possesses high thermal conductivity^28^,^31^,^36^,^37^. It is worthwhile to note that the above models are solely hypotheses. However, these hypotheses are based on the reported results of resistive memory (ReRAM) as the memristor and ReRAM may share the similar switching mechanism^38^. We will continue to investigate the real time filament formation process using advanced microscopy system in the future. In summary, the findings reported in this work unveil a strong dependence of *I*_comp_ for the electroforming process and the subsequent analog switching behavior, which will provide a useful guideline for engineering of memristor and neuromorphic systems.

**Figure 4:**
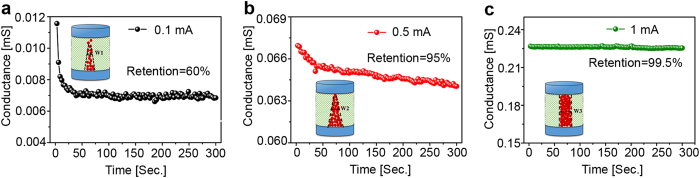
The data stability of FeO_x_ based memristor after electroforming by different *I*_comp_. The resistances read at 0.1 V bias with the duration of 3 seconds for 100 cycles after forming with I_comp_ of (**a**) 0.1 mA, (**b**) 0.5 mA, and (**c**) 1 mA, respectively.

To investigate the dynamic properties of different CFs, the conductance of memristor after electroforming was continuously read using a bias of 0.1 V for 100 cycles as shown in Fig. 4. Firstly, it is found that the conductance decayed sharply from the initial state and gradually saturates after electroforming by *I*_comp_ of 0.1 mA, indicating an unstable CF. The decay of conductance becomes moderate and is negligible for *I*_comp_ of 0.5 and 1.0 mA, respectively. After 100 cycles, the conductance reduces to 60%, 95%, and 99.5% of the original value for *I*_comp_ of 0.1, 0.5, and 1.0 mA. In addition, it is noticed that the initial spontaneous decay of the conductance is gradually inhibited by increasing *I*_comp_. It can be concluded that the stability of the CF formed in the electroforming process is able to be controlled by setting *I*_comp_ properly. Small *I*_comp_ would generate relatively less oxygen vacancies and construct pyramidal CF (inset in Fig. 4(a)) with poor data stability, while moderate *I*_comp_ would generate appropriate oxygen vacancies to construct a conical CF (inset in Fig. 4(b)) with improved data stability. However, the memristor will lose analog behavior if the *I*_comp_ is too large (inset in Fig. 4(c)) due to the formation of a strong cylindrical CF, which is not easily ruptured by joule heating. Therefore, a trade-off exists between the analog behavior and data stability of memristors.

**Figure 5:**
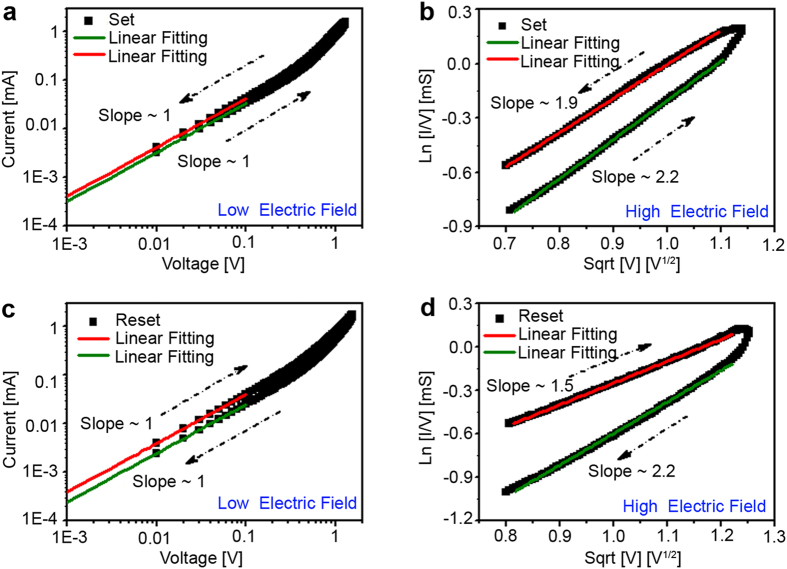
Typical analog *I*-*V* curves and fittings. (**a**) The double logarithmic graph of *I*-*V* curve of set process and linear fitting at low electric field (0 ~ 0.10 V). (**b**) *Ln*(*I*/*V*) vs. 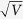 plot of set process at high electric field (0.70 ~ 1.30 V). (**c**) The double logarithmic graph of *I*-*V* curve of reset process as well as linear fitting at low electric field (0 ~ 0.10 V). (**d**) *Ln*(*I*/*V*) vs. 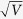 plot of reset process at high electric field (0.80 ~ 1.55 V).

To further investigate the charge transportation in FeO_x_ based memristors, *I*-*V* curves were obtained from the device formed by the moderate *I*_comp_ of 0.5 mA. Figure 5(a) displays the *I*-*V* curve of set process replotted in a dual logarithmic graph. The linear fitting of the curve at the low voltage range (0 ~ 0.10 V) has a slope of 1, indicating that ohmic conduction dominates at low electric field. The equation for ohmic conduction can be expressed as,


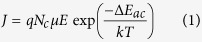


where, *q* is the electronic charge, 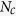 is the density of states of conduction band, μ is the mobility of the carrier, *E* is the applied electric field, 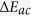 is the activation energy of electron, *k* is Boltzmann’s constant, and *T* is the temperature.

For the charge transport process at high electric field, there are four main conduction mechanisms including Schottky emission (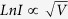), space charge-limited conduction 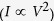, Fowler-Nordheim tunneling 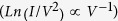, and Poole-Frenkel (P-F) emission 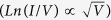
16,39. Figure 5(b) shows the *Ln*(*I*/*V*) versus 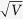 plot of the set process using positive DC. P-F emission was found to best fit the results, which indicates that P-F emission dominates the charge transport process at high electric field. The equation for P-F emission is given as,


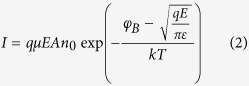


where, A is the area of the device, 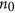 is the defect concentration, and 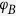 is the depth of trap from conduction band of FeO_x_ which is corrected for the electric field in the exponential way, and 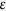 is the dynamic permittivity. The temperature-dependent resistance measurement at the high resistance state further confirms that the P-F emission accounts for the charge transport process within the FeO_x_ layer as shown in Fig. S2. Furthermore, the charge transport mechanism is explored for the reset process in which ohmic conduction (Fig. 5(c)) and P-F emission (Fig. 5(d)) dominate for the memristor at low and high electric field, respectively.

**Figure 6:**
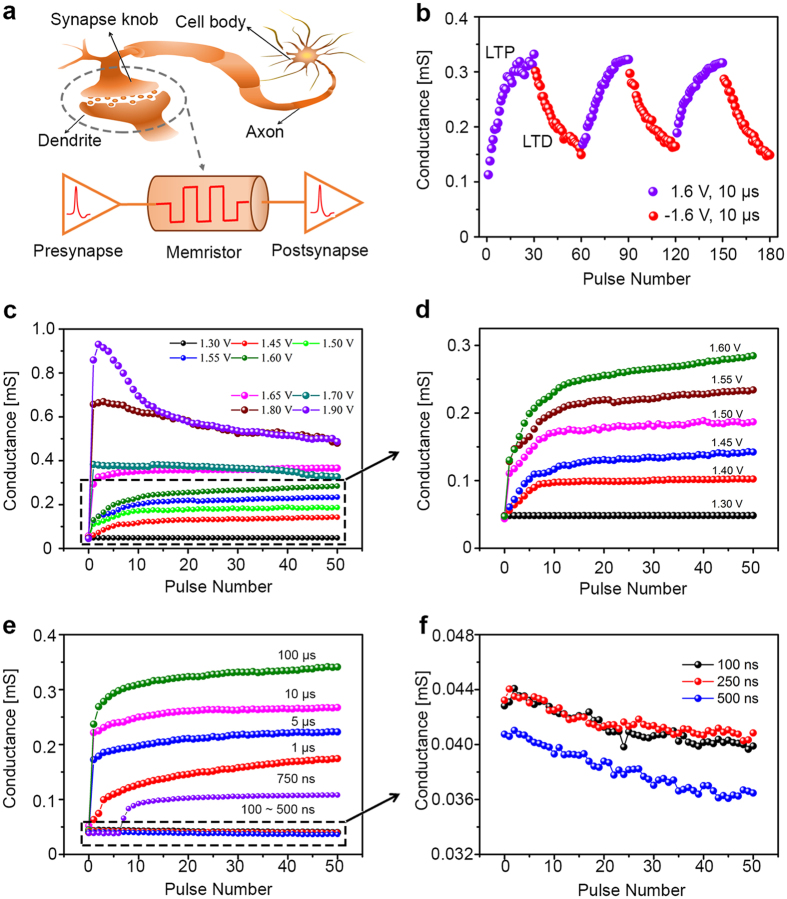
Implementation of long-term potentiation (LTP) and long-term depression (LTD) and pulse training performance on FeO_x_ based memristor. (**a**) Schematic illustration of using a memristor to emulate synapse function in neural networks. (**b**) Observation of long-term potentiation (LTP) and long-term depression (LTD) under 30 positive pulses (1.60 V, 10 μs) and 30 negative pulses (−1.60 V, 10 μs), respectively. (**c**) Pulse amplitude-dependent conductance with 50 set pulses, pulse width fixed at 10 μs and pulse interval is 1 s, pulse amplitude varies from 1.30 V to 1.90 V, (**d**) Pulse amplitude-dependent conductance with pulse width fixed at 10 μs and amplitude varied from 1.30 V to 1.60 V. (**e**) Pulse width-dependent conductance with 50 set pulses, the pulse amplitude fixed at 1.60 V, the pulse interval is 1 s and pulse width varies from 100 ns to 100 μs. (**f**) The zoom-in display of the conductance against the short pulse width from 100 ns to 500 ns with fixed 1.60 V amplitude.

To integrate the memristor as the electronic synapse in neuromorphic systems, besides the demonstration of spike-timing dependent plasticity (STDP) learning rule, the training characteristics by pulse train are also very important. However, it was neglected in the previous studies^12^,^14^,^31^,^40^,^41^. Therefore, we investigated the training characteristics by pulse train input in this work. It is widely accepted that learning and memorization process is associated with the synaptic weight modification in biology as shown in Fig. 6(a), and the synaptic weight can be modified by temporally correlated pre- and post-synaptic spiking via STDP^3^. Figure 6(b) manifests that the conductance of memristor can be successfully potentiated by thirty positive pulses and depressed by the subsequent thirty negative pulses. The retention of the memristor shown in Fig. S3 demonstrates that the conductance state could be retained in the long term timescale. These long-term potentiation (LTP) and long-term depression (LTD) characteristics can be applied to emulate the synapse plasticity of human brain^3^,^4^,^10^. In addition, the pulse training performance as a function of pulse amplitude and width was further investigated. Figure 6(c) shows the pulse amplitude dependence using 50 training pulses with a fixed width of 10 μs. Depending on the pulse amplitude, three kinds of analog behaviors are observed with various conductance changing trends. When the pulse amplitude ranges from 1.40 V to 1.60 V, the memristor conductance gradually increases in a monotonic fashion as the pulse number increases, where a zoom-in plot is shown in Fig. 6(d). When pulse amplitude increases to 1.65 V or 1.70 V, a plateau of conductance is observed following the initial jump generated by the first pulse. When the pulse amplitude is above 1.80 V, an impulse-like shape in conductance change is observed. In the first few pulses, the conductance increases dramatically. After reaching a peak, the conductance inversely decreases as the pulse number continuously increases. The variation of conductance changing behavior at different voltages is expected to relate to joule heating and electric field. In general, joule heating generated by the current enhances the drift and diffusion of oxygen vacancies^8^,^42^, while electric field introduced by pulse voltage bias induces the drift of oxygen vacancies along the direction of electric field. As voltage amplitude increases, electric field and joule heating may reach a balance causing a dynamic equilibrium of oxygen vacancies after an initial conductance jump. Hence, the conductance may remain the same, as shown in the plateau region. Below this voltage amplitude, the effect of electric field dominates the conductance changing behavior leading to a monotonic increase of conductance. Above this voltage amplitude, in the first few pulses, the large electric field drives the oxygen vacancies to form the cylinder-shaped filament as shown in Fig. 3(c). Once filament is formed, the current is significantly increased and joule heating dominates the movement of oxygen vacancies. Because of the high thermal conductivity of metal electrodes, the heat is expected to accumulate at the middle of filament and drives the oxygen vacancies moving away from the filament, resulting in a decrease of the conductance. These different analog behaviors could enhance the learning capabilities of memristor as electronic synapse to emulate comprehensive biological learning rules. When pulse amplitude is 1.30 V, no obvious conductance change is observed as shown in Fig. S4, which suggests the movement of oxygen vacancies is insufficient to influence the conductance.

To examine the effect of pulse width on memristors, pulses with amplitude of 1.60 V were applied since it provided a relatively large conductance window as observed in Fig. 6(d). The pulse width varied from 20 ns to 100 μs. The conductance increases with the increase of pulse number whilst the pulse width increases from 750 ns to 100 μs as shown in Fig. 6(e). It is found that more number of pulses is required to trigger the increment of the conductance when pulse width reduces, such as 8 pulses are required to induce the conductance increment for pulse width of 750 ns and only 3 pulses are required for pulse width of 1 μs. However, when the pulse width is shorter than 750 ns, such as 100 ns, 250 ns, and 500 ns in Fig. 6(f), the conductance slightly decreases as the pulse number increases, indicating the accumulation of heat between adjacent pulse training cycles^8^,^22^,^42^. Furthermore, it is noticed that there is also an abrupt change of conductance at the beginning of pulse training when the pulse is 1000 μs (Fig. S5). This phenomenon is possibly due to more oxygen vacancy drift driven by electric field during the training with long pulse width, leading to a large conductance change. The phenomenon shown in Fig. 6(e,f) may be utilized to emulate some other brain behaviors, such as habituation or sensitization^14^,^17^,^18^.

In addition, the energy consumption is regarded as the most important issue for the neuromorphic devices. The memristors are desired to consume energy as low as possible. In our experiments, we find that operational voltages and currents are closely relevant to the *I*_comp_. As shown in Fig. 2, the lower *I*_comp_ results in lower energy consumption and the lowest energy consumption exhibits at the lowest *I*_comp_ of 0.1 mA. However, this low *I*_comp_ may generate the pyramidal shape conductive filament, which is unstable and easy to be dissolved. Therefore, there exists a tradeoff between the energy consumption and the stability of the memory.

## Conclusion

In this work, we investigated the effect of the compliance current of electroforming process on the analog behavior using FeO_x_ based memristor. It was found that small *I*_comp_ tends to form a pyramidal CF resulting in poor data stability and the overlap of analog *I-V* curves, while large *I*_comp_ tends to form a cylindrical CF resulting in good data stability but a loss of analog behavior. *I*_comp_ with a proper value tends to form a conical CF leading to a large conductance window, good stability, and low voltage analog switching. A conductive filament model is introduced to explain the effect of *I*_comp_ on the formation of different CFs. The conduction mechanism is further investigated, which shows that ohmic conduction dominates the electron movement at low electric field and Poole-Frenkel emission accounts for its electron transport at high electric field. The LTP and LTD behaviors of FeO_x_ based memristor were demonstrated, showing the potential application in bio-inspired neuromorphic systems. Also of importance is that, the study on the pulse amplitude/width-dependence shows there are three various analog behaviors, where the conductance may monotonically increase, become plateau after initial jump, and continuously decrease after a peak, due to the competition between joule heating-induced migration and electric field-induced drift. Such different analog behaviors may enhance the learning capabilities of memristor as an electronic synapse to emulate comprehensive biological learning rules. This work would provide an important guidance in developing memristive nanodevices and the application in bio-inspired neuromorphic systems.

## Methods

### Device Fabrication

To fabricate the Pt/FeO_x_/Pt memristor devices, silicon wafer with 1 μm SiO_2_ was used as the substrate. A 100 nm thick metallic Titanium (Ti) serving as an adhesion layer was deposited on the substrate by radio frequency (RF) magnetic sputtering, then a 40 nm thick platinum (Pt) was deposited by e-beam evaporation as the bottom electrode. Subsequently, an 80-nm-thick SiO_2_ layer serving as an insulating layer was also deposited by e-beam evaporation. The 30 nm FeO_x_ switching layer was deposited by RF magnetic sputtering. Finally top electrode consisting of 40 nm Pt and 100 nm Ti was deposited by e-beam evaporation.

### Electrical Measurement

All the electrical characteristics were measured by a four-probe system (Cascade S300) equipped with a semiconductor characterization system (Keithley 4200-SCS). The various pulse conditions utilized in the testing are generated by Keithley 4200. During the electrical measurements, the positive bias was defined by the current flowing from the top electrode to the bottom one. All measurements were performed at room temperature in the air.

## Additional Information

**How to cite this article**: Wang, C. *et al.* Investigation and Manipulation of Different Analog Behaviors of Memristor as Electronic Synapse for Neuromorphic Applications. *Sci. Rep.* **6**, 22970; doi: 10.1038/srep22970 (2016).

## Supplementary Material

Supplementary Information

## References

[b1] Mead C. Neuromorphic electronic systems. Proc IEEE 78, 1629–1636 (1990).

[b2] Douglas R., Mahowald M. & Mead C. Neuromorphic analogue VLSI. Annu Rev Neurosci 18, 255–281 (1995). 7605063 10.1146/annurev.ne.18.030195.001351

[b3] Saïghi S. *et al.* Plasticity in memristive devices for spiking neural networks. Front Neurosci 9, (2015), 10.3389/fnins.2015.00051. PMC4345885 25784849

[b4] He W. *et al.* Enabling an integrated rate-temporal learning scheme on memristor. Sci Rep 4, (2014), 10.1038/srep04755. PMC3996481 24755608

[b5] Mandal S., El-Amin A., Alexander K., Rajendran B. & Jha R. Novel synaptic memory device for neuromorphic computing. Sci Rep 4, (2014), 10.1038/srep05333. PMC4061545 24939247

[b6] Strukov D. B., Snider G. S., Stewart D. R. & Williams R. S. The missing memristor found. Nature 453, 80–83 (2008). 18451858 10.1038/nature06932

[b7] Prezioso M., Merrikh-Bayat F., Hoskins B. D., Adam G. C., Likharev K. K. & Strukov D. B. Training and operation of an integrated neuromorphic network based on metal-oxide memristors. Nature 521, 61–64 (2015). 25951284 10.1038/nature14441

[b8] Kim S., Du C., Sheridan P., Ma W., Choi S. & Lu W. D. Experimental demonstration of a second-order memristor and its ability to biorealistically implement synaptic plasticity. Nano Lett 15, 2203–2211 (2015). 25710872 10.1021/acs.nanolett.5b00697

[b9] Zhu L. Q., Wan C. J., Guo L. Q., Shi Y. & Wan Q. Artificial synapse network on inorganic proton conductor for neuromorphic systems. Nat Commun 5, (2014), 10.1038/ncomms4158. 24452193

[b10] Kim K., Chen C. L., Truong Q., Shen A. M. & Chen Y. A carbon nanotube synapse with dynamic logic and learning. Adv Mater 25, 1693–1698 (2013). 23281020 10.1002/adma.201203116

[b11] Wang Y.-F., Lin Y.-C., Wang I. T., Lin T.-P. & Hou T.-H. Characterization and modeling of nonfilamentary Ta/TaO_x_/TiO_2_/Ti analog synaptic device. Sci Rep 5, (2015), 10.1038/srep10150. PMC4424833 25955658

[b12] Wang Z. Q., Xu H. Y., Li X. H., Yu H., Liu Y. C. & Zhu X. J. Synaptic learning and memory functions achieved using oxygen ion migration/diffusion in an amorphous InGaZnO memristor. Adv Funct Mater 22, 2759–2765 (2012).

[b13] Nayak A. *et al.* Controlling the synaptic plasticity of a Cu_2_S gap-type atomic switch. Adv Funct Mater 22, 3606–3613 (2012).

[b14] Ohno T., Hasegawa T., Tsuruoka T., Terabe K., Gimzewski J. K. & Aono M. Short-term plasticity and long-term potentiation mimicked in single inorganic synapses. Nat Mater 10, 591–595 (2011). 21706012 10.1038/nmat3054

[b15] Jo S. H., Chang T., Ebong I., Bhadviya B. B., Mazumder P. & Lu W. Nanoscale memristor device as synapse in neuromorphic systems. Nano lett 10, 1297–1301 (2010). 20192230 10.1021/nl904092h

[b16] Li Y. *et al.* Ultrafast synaptic events in a chalcogenide memristor. Sci Rep 3, (2013), 10.1038/srep01619. PMC3619133 23563810

[b17] Chang T., Jo S.-H. & Lu W. Short-term memory to long-term memory transition in a nanoscale memristor. ACS nano 5, 7669–7676 (2011). 21861506 10.1021/nn202983n

[b18] Wan C. J., Zhu L. Q., Zhou J. M., Shi Y. & Wan Q. Memory and learning behaviors mimicked in nanogranular SiO_2_-based proton conductor gated oxide-based synaptic transistors. Nanoscale 5, 10194–10199 (2013). 24056993 10.1039/c3nr02987e

[b19] Yamashita T. & Hayes P. Analysis of XPS spectra of Fe^2+^ and Fe^3+^ ions in oxide materials. Appl Surf Sci 254, 2441–2449 (2008).

[b20] Grosvenor A. P., Kobe B. A., Biesinger M. C. & McIntyre N. S. Investigation of multiplet splitting of Fe 2p XPS spectra and bonding in iron compounds. Surf Interface Anal 36, 1564–1574 (2004).

[b21] Feng L.-W. *et al.* Improvement of resistance switching characteristics in a thin FeO_x_ transition layer of TiN/SiO_2_/FeO_x_/FePt structure by rapid annealing. Appl Phys Lett 96, 222108 (2010), 10.1063/1.3428777.

[b22] Chen J. Y., Huang C. W., Chiu C. H., Huang Y. T. & Wu W. W. Switching kinetic of VCM‐based memristor: evolution and positioning of nanofilament. Adv Mater 27, 5028–5033 (2015). 26193454 10.1002/adma.201502758

[b23] Celano U. *et al.* Three-dimensional observation of the conductive filament in nanoscaled resistive memory devices. Nano Lett 14, 2401–2406 (2014). 24720425 10.1021/nl500049g

[b24] Park G.-S. *et al.* *In situ* observation of filamentary conducting channels in an asymmetric Ta_2_O_5−x_/TaO_2−x_ bilayer structure. Nat Commun 4, (2013), 10.1038/ncomms3382. 24008898

[b25] Wong H.-S. *et al.* Metal–oxide RRAM. Proc IEEE 100, 1951–1970 (2012).

[b26] Chen J.-Y. *et al.* Dynamic evolution of conducting nanofilament in resistive switching memories. Nano Lett 13, 3671–3677 (2013). 23855543 10.1021/nl4015638

[b27] Yang Y., Gao P., Gaba S., Chang T., Pan X. & Lu W. Observation of conducting filament growth in nanoscale resistive memories. Nat Commun 3, 732 (2012), 10.1038/ncomms1737. 22415823

[b28] Yang J. J. *et al.* The mechanism of electroforming of metal oxide memristive switches. Nanotechnology 20, 215201 (2009), 10.1088/0957-4484/20/21/215201. 19423925

[b29] Yang Y. *et al.* Electrochemical dynamics of nanoscale metallic inclusions in dielectrics. Nat Commun 5, (2014), 10.1038/ncomms5232. 24953477

[b30] Nardi F., Larentis S., Balatti S., Gilmer D. C. & Ielmini D. Resistive switching by voltage-driven ion migration in bipolar RRAM—Part I: Experimental study. Electron Devices, IEEE Transactions on 59, 2461–2467 (2012).

[b31] Kim K. M., Park T. H. & Hwang C. S. Dual conical conducting filament model in resistance switching TiO_2_ thin films. Sci Rep 5, 7844 (2015), 10.1038/srep07844. 25598439 PMC4297972

[b32] Shimeng Y. Overview of resistive switching memory (RRAM) switching mechanism and device modeling. In proceedings of *IEEE international symposium on circuits and systems (ISCAS)* Melbourne VIC, Australia, 1–5 June, 2014. pp. 2017–2020 (2014).

[b33] Li L. *et al.* Anatomy of vertical heteroepitaxial interfaces reveals the memristive mechanism in Nb_2_O_5_-NaNbO_3_ thin films. Sci Rep 5, (2015), 10.1038/srep09229. PMC4363834 25784511

[b34] Song S. J. *et al.* Real-time identification of the evolution of conducting nano-filaments in TiO_2_ thin film ReRAM. Sci Rep 3, (2013). 10.1038/srep03443. PMC3853657 24309421

[b35] Kwon D.-H. *et al.* Atomic structure of conducting nanofilaments in TiO_2_ resistive switching memory. Nat Nanotech 5, 148–153 (2010). 10.1038/nnano.2009.456 20081847

[b36] Hu C., McDaniel M. D., Posadas A., Demkov A. A., Ekerdt J. G. & Yu E. T. Highly controllable and stable quantized conductance and resistive switching mechanism in single-crystal TiO2 resistive memory on silicon. Nano Lett 14, 4360–4367 (2014). 25072099 10.1021/nl501249q

[b37] Cao X. *et al.* Effects of the compliance current on the resistive switching behavior of TiO2 thin films. Appl Phys A 97, 883–887 (2009).

[b38] Chua L. Resistance switching memories are memristors. Appl Phys A 102, 765–783 (2011).

[b39] Wu S. X. *et al.* Colossal resistance switching in Pt/BiFeO_3_/Nb:SrTiO_3_ memristor. Appl Phys A-Mater Sci Process 116, 1741–1745 (2014).

[b40] Hasegawa T. *et al.* Learning abilities achieved by a single solid-state atomic switch. Adv Mater 22, 1831–1834 (2010). 20512956 10.1002/adma.200903680

[b41] Liu D., Cheng H., Zhu X., Wang G. & Wang N. Analog memristors based on thickening/thinning of Ag nanofilaments in amorphous manganite thin films. ACS Appl Mater Interfaces 5, 11258–11264 (2013). 24083960 10.1021/am403497y

[b42] Kim S., Choi S., Lee J. & Lu W. D. Tuning Resistive Switching Characteristics of Tantalum Oxide Memristors through Si Doping. ACS Nano 8, 10262–10269 (2014). 25255038 10.1021/nn503464q

