# Supplementary material for: Investigation and Manipulation of Different Analog Behaviors of Memristor as Electronic Synapse for Neuromorphic Applications
Source: Sci Rep. 2016 Mar 14;6:22970. doi: 10.1038/srep22970 (PMC4789640; doi:10.1038/srep22970)
Supplement: Supplementary Information [file srep22970-s1.doc]

*Supplementary Information*

**Investigation and Manipulation of Different Analog Behaviors of Memristor as Electronic Synapse for Neuromorphic Applications**

Changhong Wang^†^, Wei He^†^, Yi Tong* & Rong Zhao^*^

Engineering Product Development, Singapore University of Technology & Design, 8 Somapah Road, Singapore 487372.

Correspondence and requests for materials should be addressed to R. Z. (email: [zhao_rong@sutd.edu.sg](mailto:zhao_rong@sutd.edu.sg)) or Y. Tong (email: yi_tong@sutd.edu.sg)

†Authors contribute equally to this work.

**Supplementary Results.**

**Figure S1**. **X-ray diffraction patterns of iron oxide film on the silicon wafer with the thickness of 30 nm and 60 nm, respectively.** Here two peaks shown in 30 nm FeO_x_ films maybe attribute to Fe_2_O_3_ (222) and Fe (200), causing by the epitaxial growth on silicon wafer (1 μm SiO_2_) surface. The intensity of these two peaks are dramatically depressed in the case of 60 nm, further indicating that these two peaks mainly results from the bottom of the film. The peaks around 69
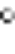
 is a typical peaks of silicon wafer (100). There are some broad peaks, demonstrating that FeO_x_ thin film is amorphous.


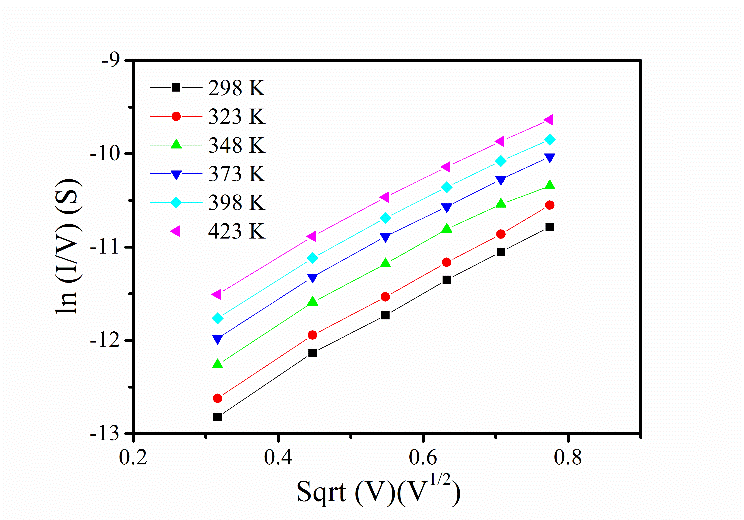


**Figure S2**. **Temperature dependence of FeO_x_ based memristor.** All the data we tested after reset process. Readout bias varies from 0.1 V to 0.6 V and testing temperature changes from 298 K, 323 K, 348 K, 373 K, 398 K, and 423 K. The linear relationship between Ln (*I*/*V*) and sqrt (*V*) indicates that P-F emission dominates charge transport process within iron oxide dielectric layer.


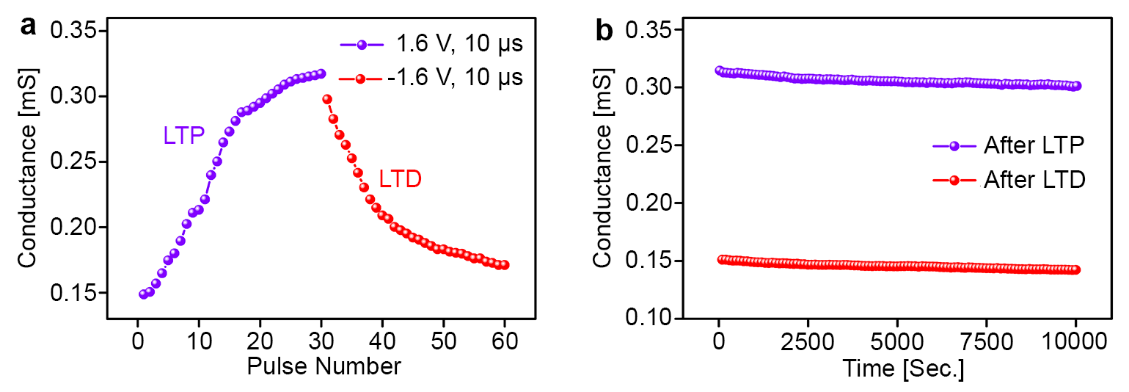


**Figure S3**. **The long-term potentiation (LTP) and long-term depression (LTD) of the FeO_x_ based memristor under 30 positive pulses (1.60 V, 10 µs) and 30 negative pulses (-1.60 V, 10 µs), respectively.** After 30 consecutive positive pulses (1.60 V, 10 μs), the data retention was recorded in Figure (b) in violet color, which shows that the conductance can be kept in a long term scale. After that, we continually did the depression process as shown in Figure (a) in red color and the data retention was recorded in Figure (b) in record color, which also illustrates that the conductance can be kept in the long term scale. Based on above analysis, we claim FeO_x_ based memristor shows LTP and LTD, which can emulate the plasticity of human brain.


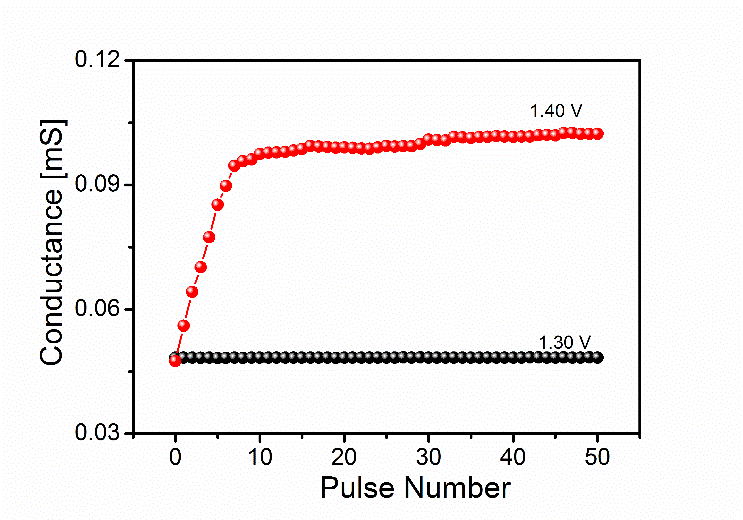


**Figure S4**. **The conductance dependence of the FeO_x_ based memristor on the amplitude of 1.3V and the width of 10 μs.** Here we can clearly see there is no obvious changes in the conductance after 50 pulses (1.3 V, 10 μs), but the conductance can be gradually increased on the amplitude of 1.4 V, which indicates that the threshold pulse amplitude is about 1.3 V when the pulse duration is 10 μs.


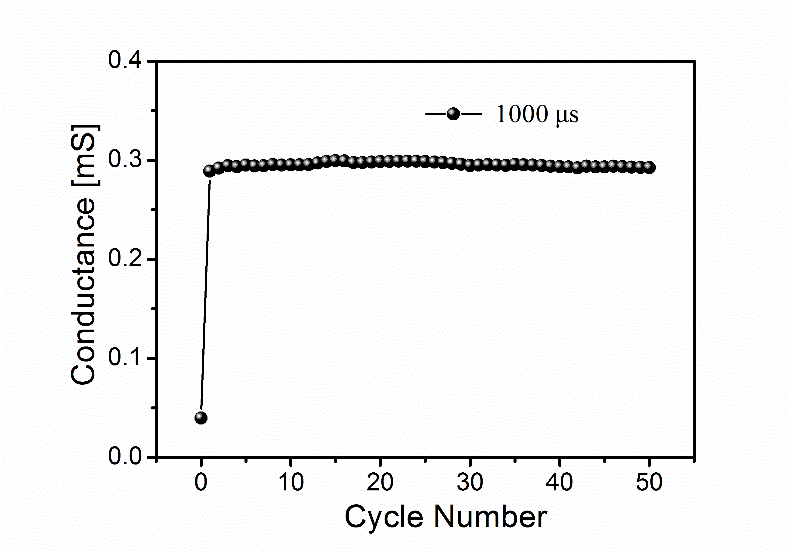


**Figure S5**. **The conductance dependence of the FeO_x_ based memristor on the pulse amplitude of 1.6 V and the width of 1000 μs.** After first abrupt switching, the conductance almost keeps stable in the following pulse training, indicating that electric field and joule heating reach a balance under this situation.
